# Quantification of DNA by a Thermal-Durable Biosensor Modified with Conductive Poly(3,4-ethylenedioxythiophene)

**DOI:** 10.3390/s18113684

**Published:** 2018-10-30

**Authors:** Yesong Gu, Po-Yuan Tseng, Xiang Bi, Jason H. C. Yang

**Affiliations:** 1 Department of Chemical and Materials Engineering, Tunghai University, Taichung 40704, Taiwan; tseng1595@gmail.com (P.-Y.T.); g06310801@thu.edu.tw (X.B.); 2 Department of Fiber and Composite Materials, Feng Chia University, Taichung 40724, Taiwan; jasonhcyang@gmail.com

**Keywords:** poly(3,4-ethylenedioxythiophene), DNA biosensor, gold nanoparticle, electron transfer mediate

## Abstract

The general clinical procedure for viral DNA detection or gene mutation diagnosis following polymerase chain reaction (PCR) often involves gel electrophoresis and DNA sequencing, which is usually time-consuming. In this study, we have proposed a facile strategy to construct a DNA biosensor, in which the platinum electrode was modified with a dual-film of electrochemically synthesized poly(3,4-ethylenedioxythiophene) (PEDOT) resulting in immobilized gold nanoparticles, with the gold nanoparticles easily immobilized in a uniform distribution. The DNA probe labeled with a SH group was then assembled to the fabricated electrode and employed to capture the target DNA based on the complementary sequence. The hybridization efficiency was evaluated with differential pulse voltammetry (DPV) in the presence of daunorubicin hydrochloride. Our results demonstrated that the peak current in DPV exhibited a linear correlation the concentration of target DNA that was complementary to the probe DNA. Moreover, the electrode could be reused by heating denaturation and re-hybridization, which only brought slight signal decay. In addition, the addition of the oxidized form of nicotinamide adenine dinucleotide (NAD^+^) could dramatically enhance the sensitivity by more than 5.45-fold, and the limit-of-detection reached about 100 pM.

## 1. Introduction

Recent research in DNA biosensor fabrication has gained remarkable progress by offering reliable approaches to quantify DNA and detect mutations [1,2,3]. A DNA biosensor is generally constructed according to the specific complementarity between a specific target DNA and a designed probe DNA [4,5]. In order to make it easy to capture the probe DNA, several approaches have been proposed, such as employing gold nanoparticles (Au NPs) to bridge the functionalized groups on the surface of electrode and the SH group attached to a DNA probe [6,7,8]. Nevertheless, modification of the electrode with various functional groups requires a tedious chemical process.

Coating the electrode with conductive polymer, such as polyaniline or PEDOT, has become a prospective strategy to construct a biosensor for DNA quantification because the conductive film creates not only a unique surface morphology to immobilize the DNA probe but also has excellent electrochemical aspects for detection [9,10]. Among them, PEDOT is superior to other conductive polymers due to its great environmental and thermal stabilities [11,12]. PEDOT may achieve even higher conductivity by a proper doping that could promote electron transfer, making it a suitable mean for electrochemical biosensors [13,14]. To avoid the traditional chemical crosslinking procedure, Au NPs have also been proposed in the PEDOT modified DNA biosensor through a chemical approach or an embedment method [15,16]. However, those methods could not ensure the unique distribution of the probe DNA on PEDOT. 

In this study, we often observed the PEDOT film fracturing during its electrochemical synthesis on a Pt wire as well as following measurements, which significantly influenced its performance. Therefore, a novel strategy is proposed for constructing a DNA biosensor by establishing a thermally stable dual-layer PEDOT film that can easily capture Au NPs for probe DNA immobilization. The methods for target DNA hybridization and detection with differential pulse voltammetry (DPV) were similar to our previous report [17]. The newly prepared electrode, however, demonstrated dramatic improvements in durability and thermal stability, and its sensitivity was further enhanced by employing oxidative nicotinamide adenine dinucleotide (NAD^+^). In order to simplify the clinical procedure for viral DNA detection or gene mutation diagnosis following polymerase chain reaction (PCR), it is expected the thermal-durable DNA biosensor for monitoring the DNA amplification would be relatively sensitive, fast, affordable, and involve less chemicals [18,19,20,21].

**Figure 1:**
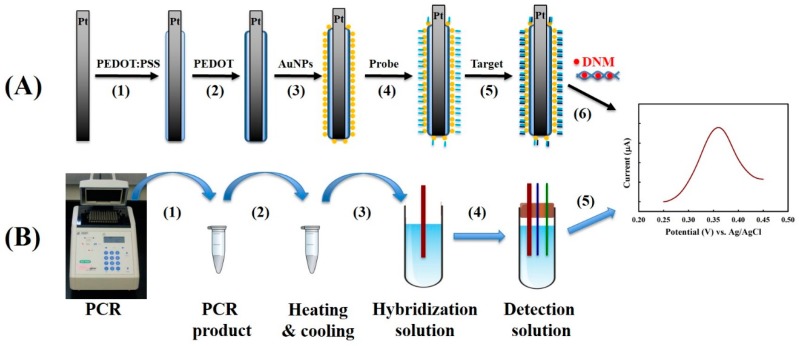
(**A**) Procedure for DNA biosensor construction: (1) synthesis of the first layer PEDOT film using poly(styrenesulfonic acid sodium salt) as the template (named as PEDOT:PSS), (2) synthesis of the second layer PEDOT film, (3) attachment of Au NPs, (4) immobilization of probe DNA, (5) hybridization of target DNA, (6) daunorubicin hydrochloride (DNM) intercalation and electrochemical detection. (**B**) Proposed scheme for PCR product detection: (1) PCR reaction, (2) heating and cooling procedure in the presence of competitive oligo in order to obtain the single strand target DNA, (3) DNA hybridization of target DNA with the probe DNA, (4) assembling the miniature electrochemical cell, and (5) electrochemical detection. The right figure indicates the DPV profile of working electrode in response to the concentration of target DNA.

We have proposed a facile strategy to construct the DNA biosensor (Figure 1A) based on our previous success in rapid PCR product response by a DNA biosensor with a heating and cooling procedure in the presence of a specific competitive oligo (Figure 1B). The proposed DNA biosensor is thermally durable in a way that is far superior to many glass carbon or disc electrode DNA biosensors, which has the potential to replace gel electrophoresis and DNA sequencing for PCR product analysis in the future.

## 2. Materials and Methods

### 2.1. Chemicals

3,4-ethylenedioxythiophene (EDOT) and daunorubicin hydrochloride (DNM) were obtained from Sigma-Aldrich Corp. at St. Louis, MO in USA. Poly(styrenesulfonic acid sodium salt) (PSS, MW 70,000) was purchased from Alfa Aesar at Heysham in United Kingdom. Au NPs with an average size of about 16 nm were homemade by reducing gold chloride in a sodium citrate aqueous solution according to literature [22]. All other reagents employed in this study were of analytical grade from various commercial sources. 

### 2.2. Pt Electrode Modification

A Pt wire with the diameter of 0.6 mm was polished in a sequential steps and followed by drying in a nitrogen purge as described in our previous publication [17]. It was then served as a working electrode with a counter electrode (a Pt wire) and a reference electrode (an Ag/AgCl in 3 M NaCl) in a miniature electrochemical cell. In 50 mM LiClO_4_ solution containing 5 mM of EDOT and 1.5 mM of PSS under 25 °C, EDOT was electrochemically polymerized on the Pt working electrode by cyclic voltammetry (CV) technique using a CHI621B electrochemical analyzer (CH Instruments, Austin, TX, USA), where the potential window was set from 0.0 to 1.0 V with a sweeping rate of 10 mV·s^−1^ for three cycles. The PEDOT:PSS/Pt electrode was rinsed with distilled water and then immersed in 100 mM LiClO_4_ solution containing 10 mM of EDOT in the absence of PSS. The second layer PEDOT was synthesized electrochemically with the same operation parameters but for two cycles only. The dual-layer PEDOT modified electrode, with an approximate total working area of 0.1913 cm^2^, was further modified with Au NPs by soaking in a 6 mL of home-made gold nanoparticle solution with 1 cm in depth for one h under 25 °C and then rinsed with distilled water. The surface morphologies of the electrodes under every preparation stages were visualized by an ABT-150S SEM (TOPCON Corp., Tokyo, Japan).

### 2.3. Designs of Probe and Target DNA 

Two DNA oligoes with sequences of 5′-CGCCGGCCACGAGAATAG-(CH)6-SH-3′ and 5′-GCTATTCTCGTGGCCGGCG-3′ served as probe and target, respectively, which have been identified as the common mutation site for *hMYH*, a gene that encodes a DNA mismatch repair ezyme, related to the hereditary non-polyposis colorectal cancer syndrome (HNPCC) [23]. The probe was capped with a -SH functional group at its 3′ terminal and the C6 chain was designed to ensure the efficiency of target DNA hybridization by eliminating possible steric hindrance. 

### 2.4. DNA Biosensor Fabrication and Performance

In order to immobilize the probe DNA to Au NPs on the PEDOT/PEDOT:PSS/Pt electrode, the working electrode was submerged in 10 mM phosphate buffered saline (PBS) buffer (pH = 7.0) containing 1 μM of probe DNA under 25 °C for 24 h and rinsed by PBS buffer. The probe DNA was then hybridized with different concentrations of target DNA in 10 mM PBS buffer (pH = 7.0) under 60 °C, followed by resining with PBS buffer. After hybridization, the working electrode was soaked in 10 mM PBS buffer (pH = 7.0) containing 10 M of DNM for 30 min under 25 °C. Finally, the working electrode was assembled in a miniature electrochemical cell. DPV was performed to characterize the hybridization efficiency in 10 mM PBS solution (pH = 7.4), where the sweeping potential window was from 0.2 to 0.7 V with optimized pulse parameters (potential: 0.01 V, width: 0.06 s, and period: 0.2 s) under room temperature.

## 3. Results and Discussion

### 3.1. Constructing an AuNP-PEDOT/Pt Electrode

**Figure 2:**
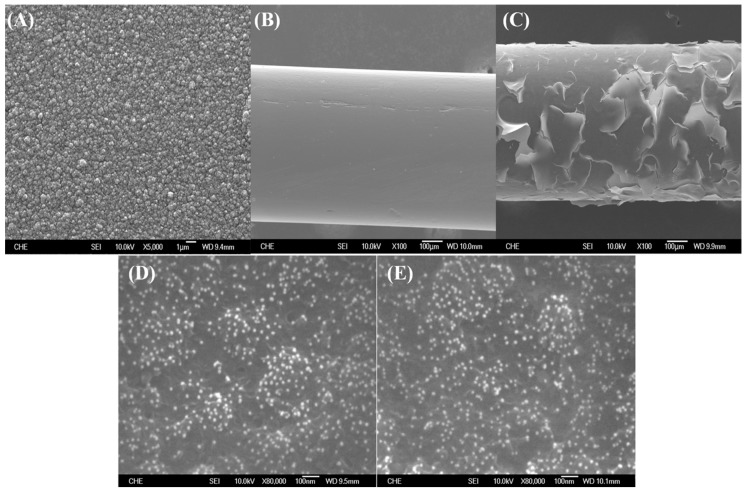
SEM analysis of the electrochemically synthesized PEDOT film on a Pt electrode at (**A**) 5000× magnification, at 100× magnification (**B**) before and (**C**) after DPV measurement (potential window: 0–1 V), and of non-cracked PEDOT films with the adsorption of Au NPs (**D**) before and (**E**) after DPV measurement.

Figure 2A,B are the topographical features of the electrochemically synthesized PEDOT films on the Pt electrode, where PEDOT forms a typical rough surface with a granular landscape. However, we have noticed that the performance of the electrode is greatly challenged by the stability of the PEDOT film, where severe cracks of PEDOT film often occur when higher potential (>1 V) or multiple measurements are applied as shown in Figure 2C. 

Generally, probe DNAs are immobilized through chemical crosslinking with the functional groups modified on the surface of the electrode, which is often considered an unreliable and inefficient procedure [17]. In order to simplify the method for the construction of a biosensor and to improve the reliability of its performance, with a theoretical belief that Au NPs are able to form stable covalent Au-S bonds with the -SH groups, we have attempted to employ Au NPs to capture the probe DNA with a thiol group at its 3′ terminal. According to Figure S1 (Supplementary Materials), our homemade Au NPs have an average diameter of about 16 nm. Zeta potential analysis indicates that the surfaces of Au NPs were negatively charged in 10 mM of PBS buffer (data not shown). Although Au NPs have been reported to be immobilized in the PEDOT film during the electrochemical synthesis, its distribution and surface unity are not satisfied [24]. In this study, we have noticed that Au NPs are able to spread across the PEDOT film by submerging the PEDOT film coated electrode in the Au NPs solution (Figure 2D), and its amount increases with increasing Au concentration as well as prolonging the process time (data not shown). The adsorbed Au NPs are confirmed by the SEM/EDS analysis (Figure S2, Supplementary Materials). The density of Au NPs within the non-cracked area remains almost unchanged after multiple DPV measurements (Figure 2E), indicating the stable interaction between Au NPs and PEDOT. It has also indicated that the stability of PEDOT film is somewhat improved by Au NPs based on the observation of reduced cracks.

### 3.2. Construction of an AuNP-PEDOT/PEDOT:PSS/Pt Electrode

Nevertheless, we still observed severe cracking of the PEDOT film during the measurements. This instability can be ascribed partially to the formation of short PEDOT fragments on the bare Pt electrode. Meanwhile, the surface of Pt electrode is rather hydrophilic, but the PEDOT film is relatively hydrophobic, which may lead to poor adhesion at the interface, especially under an extra mechanical strain. To overcome these problems of the hydrophilic PSS, an anionic polyelectrolyte as well as a common template for the synthesis of conductive PEDOT was employed in the electrochemical polymerization reaction. The SEM image of PEDOT:PSS film is shown in (Figure S3, Supplementary Materials), where the surface becomes relatively smooth. It is assumed that PSS lays down on the surface of Pt electrode and serves as a template, which ensures the PEDOT molecules stretch out instead of forming a granular landscape. The resulted PEDOT:PSS film is endurable after sequential treatments and multiple measurements, however, the Au NPs are hardly detected on the PEDOT:PSS film. This phenomenon is mainly attributed to the electric repulsion between the exposed anionic PSS and to the Au NPs being surrounded by anionic ions.

**Figure 3:**
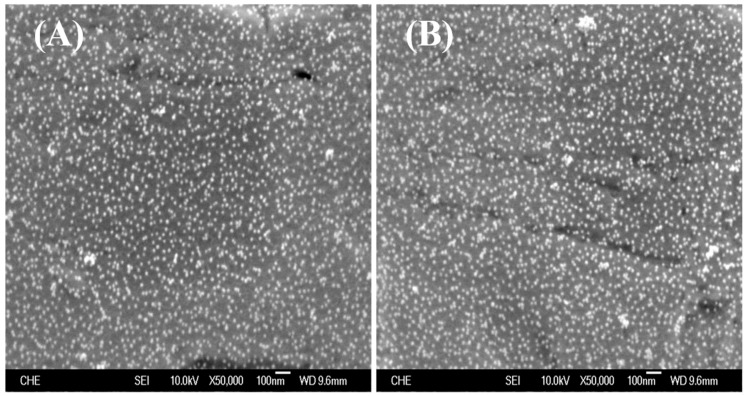
SEM topography images of PEDOT/PEDOT:PSS dual-layer film on the Pt electrode (**A**) before and (**B**) after multiple DPV measurements.

In order to achieve an ideal Au NPs immobilization, an extra electrochemical polymerization of PEDOT film is applied in the absence of PSS on the existing PEDOT:PSS film. Although the surface morphology of the dual-layer PEDOT film is similar to that of PEDOT:PSS, Au NPs could be easily attracted to the PEDOT film with a nearly uniform distribution (Figure 3A), suggesting the majority of negatively charged PSS is probably masked. Similar to our recent publication [25], the formation of strong Au-S bond between Au NPs and the sulfur in PEDOT ensured the stability of Au NPs on the PEDOT film (Figure 3B).

**Figure 4:**
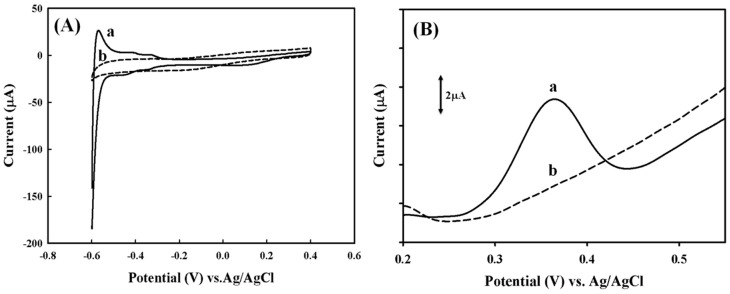
(**A**) CV profiles of (a) Pt, and (b) PEDOT/PEDOT:PSS/Pt electrodes in response to 0.1 m PBS buffer (pH = 6.2). (**B**) DPV profiles of (a) PEDOT:PSS/Pt and (b) PEDOT/PEDOT:PSS/Pt electrodes in response to 1 μM of DNM.

Figure 4A exhibits the cyclic voltammograms of bare platinum (Pt) and PEDOT/PEDOT:PSS/Pt electrodes in a 0.1 M PBS buffer (pH = 6.2) with a sweep rate of 0.2 V/s. Several redox peaks on the bare Pt electrode are observed between −0.6 and −0.3 V (line a in Figure 4A), which are attributed to the hydrogen adsorption-desorption following the electrolysis of water at the Pt electrode [9]. The overactive behavior of Pt might lead the decay of electrode stability that influences its sensitivity toward to the target molecules. By contrast, these redox peaks completely disappeared on the Pt electrode covered by the electrochemical synthesized dual-layer PEDOT film (line b in Figure 4A), indicating an effective elimination of the background interferences from the platinum by the PEDOT/PEDOT:PSS modification.

Daunorubicin hydrochloride (DMN) is an electroactive chemical that is able to be intercalated into the duplex DNA, therefore could serve as an indicator to quantify DNA [17]. Figure 4B demonstrates the blank response of PEDOT:PSS/Pt and PEDOT/PEDOT:PSS/Pt electrodes to the 1 μM DNM, where the DNM signal on the PEDOT:PSS/Pt electrode is observed (line a in Figure 4B), but not on the PEDOT/PEDOT:PSS/Pt electrode (line b in Figure 4B). The possible exposure of PSS on the surface results in PEDOT:PSS film to be negatively charged, which might attract the positively-charged DNM molecules through an electrostatic interaction. Figure 4B further verifies that the second layer of PEDOT film in the PEDOT/PEDOT:PSS/Pt electrode almost completely masks the PSS, which eliminates the interference of non-specific binding of DNM to the PEDOT film.

### 3.3. DNA Biosensor Measurement

**Figure 5:**
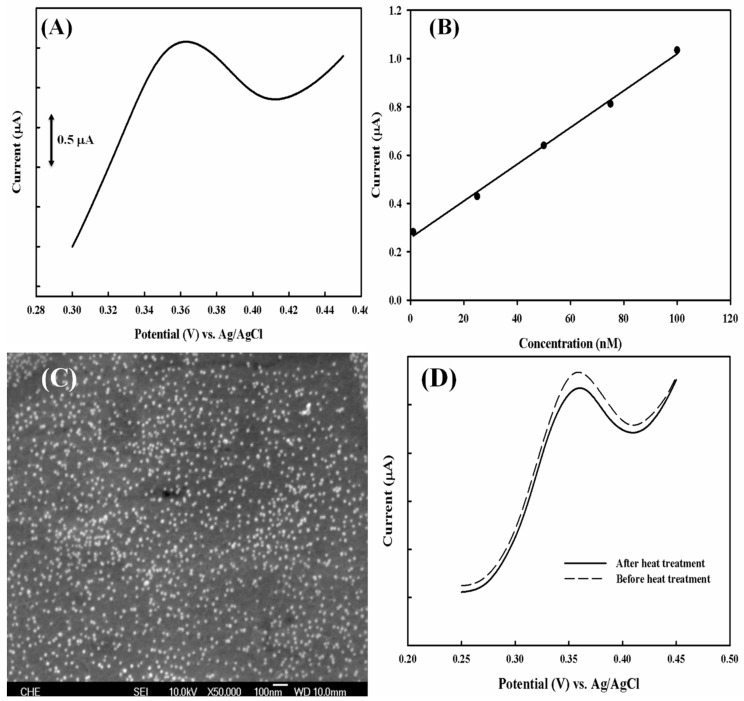
(**A**) DPV profile of ssDNA-AuNP-PEDOT/PEDOT:PSS/Pt in response to 75 nM of complementary target DNA. (**B**) The linear correlation between the peak current and the concentration of target DNA. (**C**) SEM image of ssDNA-AuNP-PEDOT/PEDOT:PSS/Pt electrode after heat treatment at 90 °C for 10 min. (**D**) DPV profiles in response to 50 nM of complementary DNA before (dotted line) and after (solid line) heat treatment.

After a successful construction of the stable PEDOT modified electrode with a lower baseline current, an 18-mer probe DNA labeled with a thiol group at its 3′ terminal was designed, which was followed by hybridization with a complementary target DNA. DNM is then intercalated into the double stranded DNA, and DPV is performed to measure the amount of DNM that corresponds to the double stranded DNA as well as the target DNA concentration in the sample solution. Figure 5A shows the DPV profiles for the response of target DNA where the peak potential is about 0.35 V, which is in good agreement with the response of DNM as shown in Figure 4. Accordingly, a linear correlation between current and target DNA is obtained with a coefficient of 0.9968 (Figure 5B). With the electrode working area of ~0.1913 cm^2^, the sensitivity of the electrode is calculated to be 39.72 nA·nM^−1^·cm^−2^.

To evaluate the performance of the electrode under multiple measurements, the hybridized DNA is separated by soaking the electrode in 90 °C PBS buffer for 10 min, followed by repetition of the hybridization of target DNA after cooling, and finally to DPV measurement with DNM. Figure 5C shows the SEM analysis of the electrode after a heating treatment. The density of Au NPs on each film is almost the same, which is approximately 98 particles/500 nm^2^, suggesting no apparent loss of Au NPs. The PEDOT film remains undamaged compared to Figure 3B. Meanwhile, the second measurement presents a similar DPV profile to the first measurement in terms of peak position and peak current (Figure 5D). The reduction of peak current is less than 6%, which provides a rather positive indication of reusability for DNA biosensor application.

**Figure 6:**
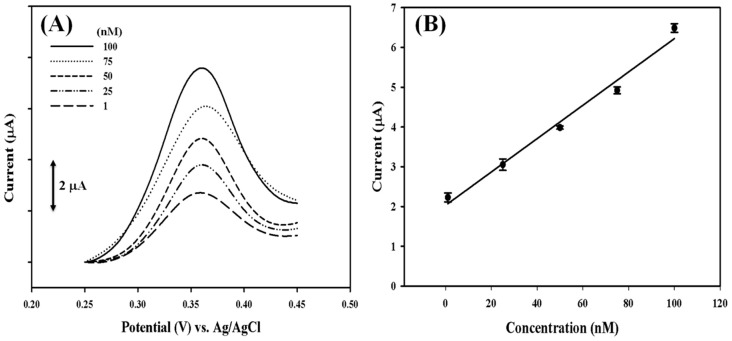
(**A**) DPV profiles of ssDNA-AuNP-PEDOT/PEDOT:PSS/Pt electrode in response to different concentrations of complementary target DNA in the presence of 10 μM of oxidized form NAD^+^. (**B**) The linear correlation between the peak current and the concentration of target DNA.

Although the current response to target DNA with a concentration below 1 nM is insignificant, it implicates an inefficient electron transfer along the DNA strand. Therefore, the possible contribution of electron transfer mediators, such as reduced and oxidized forms of NADH, were investigated. There was no notable current response found in either form of NADH in the absence of DNM. No improvement was obtained while the reduced form of NADH was employed in the presence of DNM. However, 10 μM of oxidized form NAD^+^ dramatically enhanced the DPV peak current (Figure 6A). This result indicates that electrons are transferred from the electrode to the site of DNM within the applied potential window. A linear correlation is observed with a 5.45-fold (Figure 5B) enhanced sensitivity of 216.39 nA·nM^−1^·cm^−2^ (Figure 6B). 

## 4. Conclusions

Previously, we have reported a PEDOT modified DNA biosensor that is capable of quantifying ssDNA, detecting mutations, and even the dsDNA product from a PCR reaction. With the aim of achieving real-time monitoring of DNA coupled with a PCR reaction, the DNA biosensor is expected to possess a good thermal stability in addition to ensured accuracy and extreme sensitivity. 

In this study, we have further demonstrated the advantages of the PEDOT modified biosensor in reducing background interference from the Pt electrode for the fabrication of a DNA biosensor. We have also revealed the inevitably of cracks in the PEDOT film during multiple measurements, which is possibly attributed to weak adhesion between the hydrophilic Pt electrode and the hydrophobic PEDOT film, as well as the short fragments of PEDOT polymer synthesized. We therefore employed an anionic polyelectrolyte PSS, which not only served as a perfect template for the electrochemical polymerization of PEDOT, but also acted as a bridge between the Pt electrode and the PEDOT to dramatically improve the stability of PEDOT film. Meanwhile, we discovered that our homemade Au NPs were able to easily attach to the PEDOT film with a uniform distribution, but not to the PEDOT:PSS film. We have also incorporated the addition of oxidized NAD^+^ to accelerate the electron transfer from the electrode to the site of DNM, which significantly enhanced the sensitivity of detection by more than 5.45-fold. The final calibration has demonstrated a linear correlation between the DPV peak current and the concentration of target DNA, and the limit-of-detection is about 100 pM. with a peak current of 0.254 μA based on our visual evaluation (data not shown). 

## Supplementary Materials

The following are available online at http://www.mdpi.com/1424-8220/18/11/3684/s1, Figure S1: (A) TEM image of Au NPs. (B) UV-vis absorption spectrum of Au NPs in 1% of sodium citrate solution. Figure S2: SEM/EDS analysis of the AuNPs-PEDOT/Pt electrode. Figure S3: SEM image of PEDOT:PSS single-layer film on the Pt electrode after Au NPs immobilization.
