# Supplementary material for: Quantification of DNA by a Thermal-Durable Biosensor Modified with Conductive Poly(3,4-ethylenedioxythiophene)
Source: Sensors (Basel). 2018 Oct 30;18(11):3684. doi: 10.3390/s18113684 (PMC6264125; doi:10.3390/s18113684)
Supplement: Supplementary file 1 [file sensors-18-03684-s001.pdf]

Supplementary material

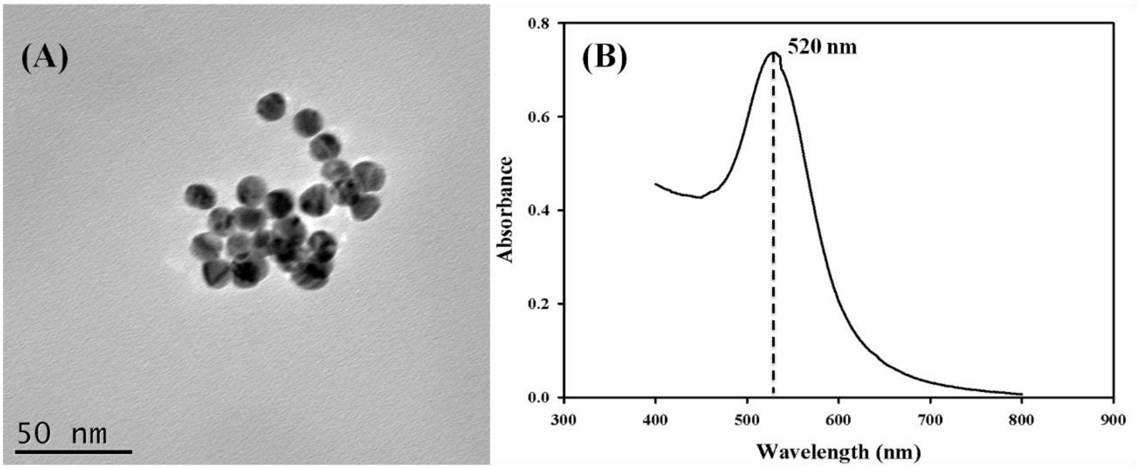

Figure S1. (A) TEM image of Au NPs. (B) UV-vis absorption spectrum of Au NPs in 1% of sodium citrate solution.

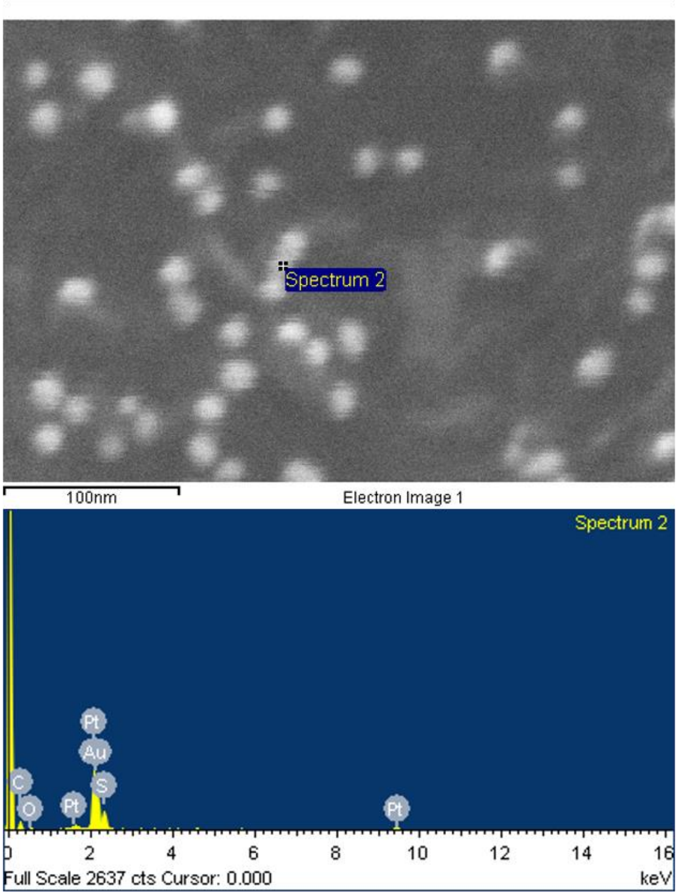

Figure S2. SEM/EDS analysis of the AuNPs-PEDOT/Pt electrode.

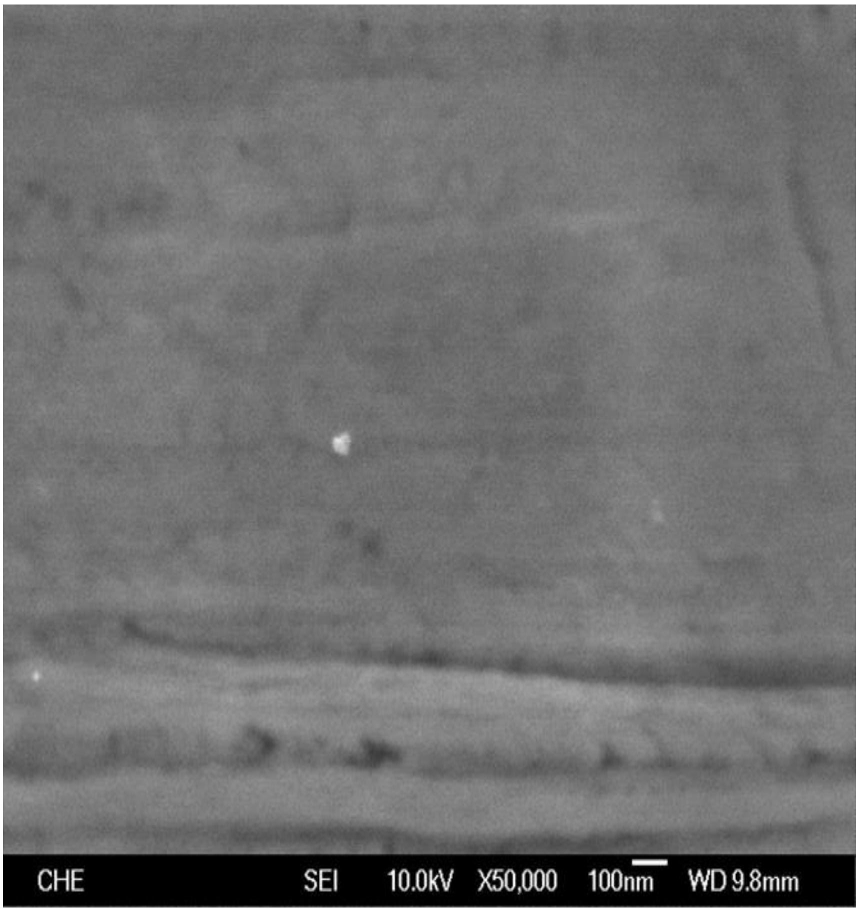

Figure S3. SEM image of PEDOT:PSS single-layer film on the Pt electrode after Au NPs immobilization.
